# Supplementary material for: Disulfides from the Brown Alga Dictyopteris Membranacea Suppress M1 Macrophage Activation by Inducing AKT and Suppressing MAPK/ERK Signaling Pathways
Source: Mar Drugs. 2020 Oct 24;18(11):527. doi: 10.3390/md18110527 (PMC7690855; doi:10.3390/md18110527)
Supplement: Supplementary file 1 [file marinedrugs-18-00527-s001.pdf]

Supplementary Materials for

Disulfides from the brown alga *Dictyopteris membranacea* suppress M1 macrophage activation by inducing AKT and suppressing MAPK/ERK signaling pathways

Maria G. Daskalaki [1,2], Paraskevi Bafiti [1], Stefanos Kikionis [3], Maria Laskou [1], Vassilios Roussis [3], Efstathia Ioannou [3], Sotirios C. Kampranis [4,*] and Christos Tsatsanis [1,2,*]

1 Laboratory of Clinical Chemistry, School of Medicine, University of Crete, Heraklion 70013, Greece; m.daskalaki@med.uoc.gr (M.G.D.); vivi.mpafiti@gmail.com (P.B.); maria.laskou95@gmail.com (M.L.)
2 Institute of Molecular Biology and Biotechnology, FORTH, Heraklion 71100, Greece
3 Section of Pharmacognosy and Chemistry of Natural Products, Department of Pharmacy, National and Kapodistrian University of Athens, Panepistimiopolis Zographou, Athens 15771, Greece; skikionis@pharm.uoa.gr (S.K.); roussis@pharm.uoa.gr (V.R.); eioannou@pharm.uoa.gr (E.I.)
4 Section of Plant Biochemistry, Department of Plant and Environmental Sciences, University of Copenhagen, Thorvaldsensvej 40, 1871 Frederiksberg, Denmark
* Correspondence: tsatsani@med.uoc.gr (C.T.); soka@plen.ku.dk (S.C.K.); Tel.: +30-2810-394833 (C.T.); +45-3131-5539 (S.C.K.)

Table S1. List of oligonucleotides used in real time PCR reactions.

Primer	Sequence
rsp9-Fwd	GCTAGACGAGAAGGATCCCC
Rsp9-Rev	CAGGCCCAGCTTAAAGACCT
Arginase 1-Fwd	CAGAAGAATGGAAGAGTCAG
Arginase 1-Rev	CAGATATGCAGGGAGTCACC
MRC1-Fwd	TTTCCATCGAGACTGCTGC
MRC1-Rev	ACCAAAGCCACTTCCCTTC
IRAK-M-Fwd	TTCCTGGCACGTTCGAATCA
IRAK-M-Rev	CGCTGCAGCAAAATCCGTTA
iNOS-Fwd	TCCTGGAGGAAGTGGGCCGAAG
iNOS-Rev	CCTCCACGGGCCCGGTACTC
TNFα-Fwd	GCCCACGTCGTAGCAAACCACC
TNFα-Rev	CGGGGCAGCCTTGTCCCTTG
Glut1-Fwd	AAAGAAGAGGGTCGGCAGAT
Glut1-Rev	CTGCCTTCTCGAAGATGCTC
Glut3-Fwd	GATCGGCTCTTTCCAGTTTG
Glut3-Rev	GATCGGCTCTTTCCAGTTTG
Pgc1α-Fwd	ACCCTGCCATTGTTAAGACC
Pgc1α-Rev	TGCTGCTGTTCCTGTTTTC
IL-6-Fwd	CAAAGCCAGAGTCCTTCAGAG
IL-6-Rev	CACTCCTTCTGTGACTCCAGC

Supplementary figures: Purity analysis of the isolated compounds

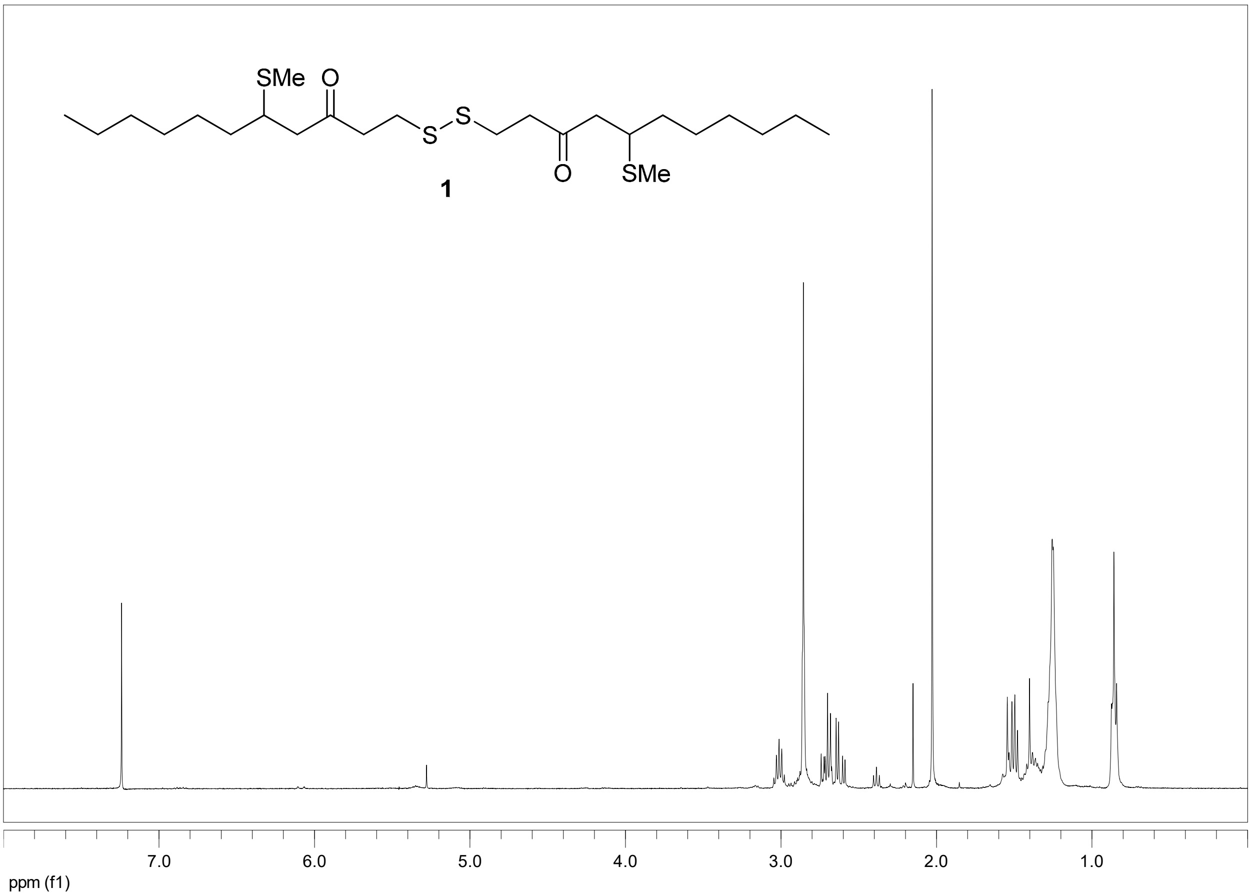

Figure S1. ^{1}H NMR spectrum (400 MHz, $CDCl_3$) of bis(5-methylthio-3-oxo-undecyl) disulfide (**1**).

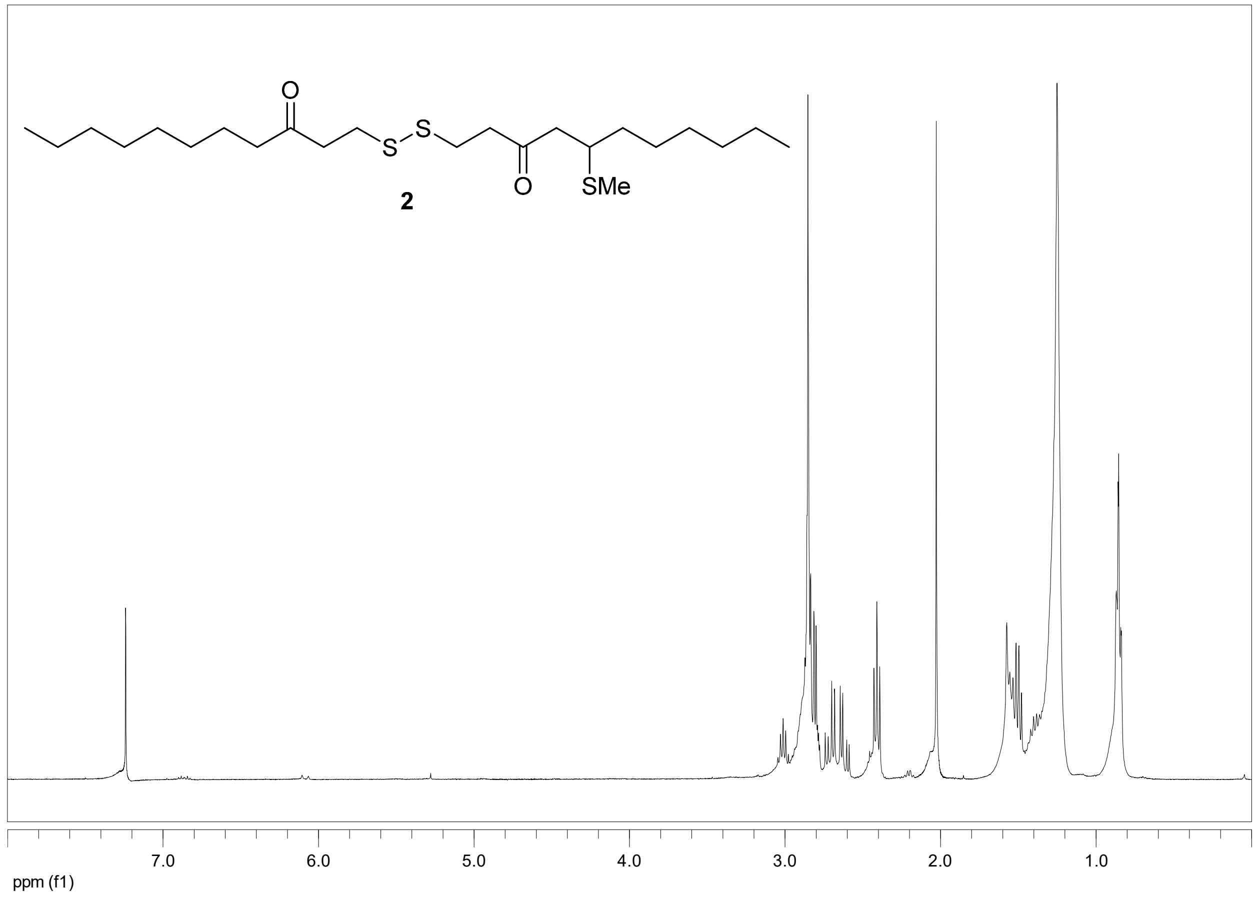

Figure S2. ^{1}H NMR spectrum (400 MHz, $CDCl_3$) of 5-methylthio-1-(3-oxo-undecyl)disulfanylundecan-3-one (**2**).

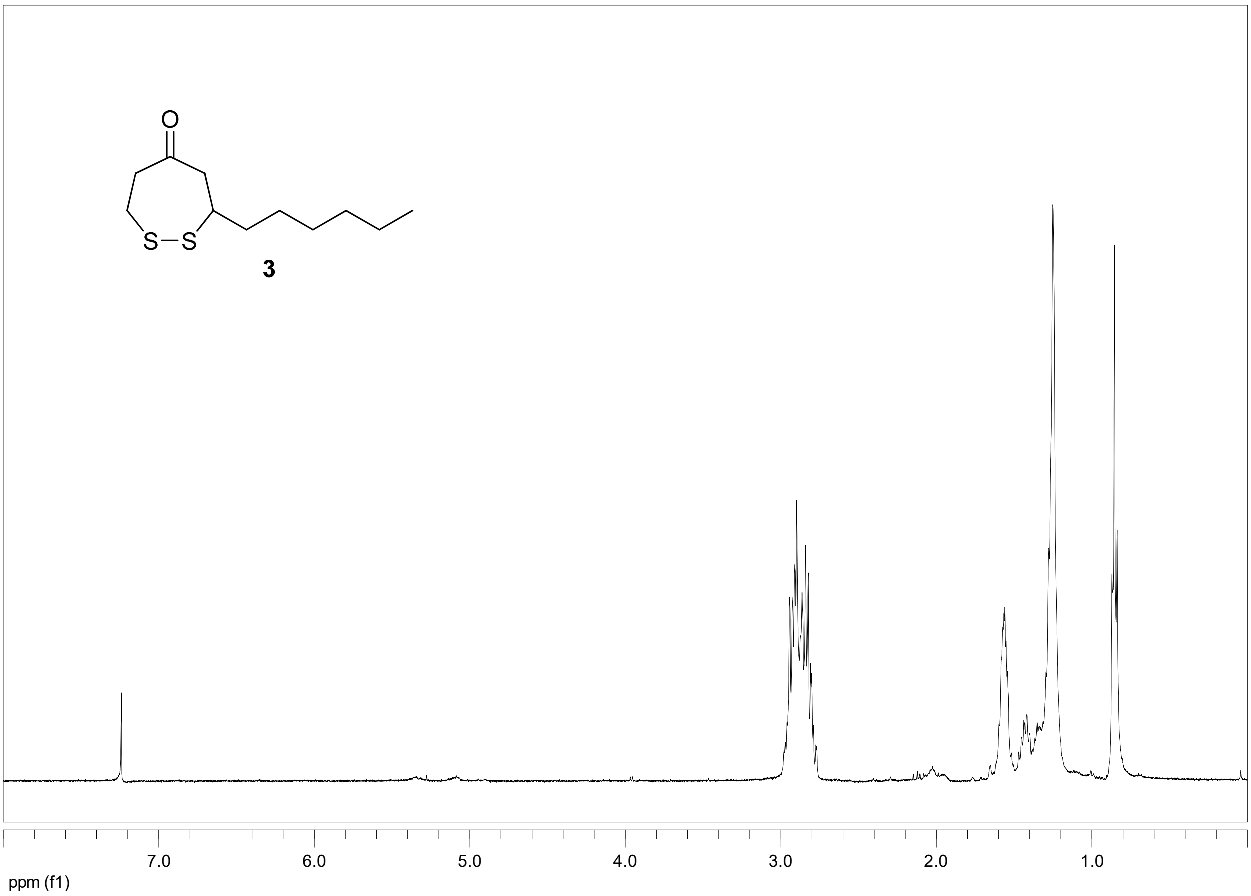

Figure S3. ^{1}H NMR spectrum (400 MHz, $CDCl_3$) of 3-hexyl-4,5-dithiocycloheptanone (**3**).
